# Mechanism of activation of porcine dendritic cells by an α-D-glucan nanoparticle adjuvant and a nanoparticle/poly(I:C) combination adjuvant

**DOI:** 10.3389/fimmu.2022.990900

**Published:** 2022-09-05

**Authors:** Juan F. Hernandez-Franco, Shaojun Xie, Jyothi Thimmapuram, Darryl Ragland, Harm HogenEsch

**Affiliations:** ^1^ Department of Comparative Pathobiology, College of Veterinary Medicine, Purdue University, West Lafayette, IN, United States; ^2^ Bioinformatics Core, Purdue University, West Lafayette, IN, United States; ^3^ Department of Veterinary Clinical Sciences, College of Veterinary Medicine, Purdue University, West Lafayette, IN, United States; ^4^ Purdue Institute of Inflammation, Immunology and Infectious Diseases, Purdue University, West Lafayette, IN, United States

**Keywords:** Vaccines, nanoparticles, adjuvants, dendritic cells, poly(I:C), transcriptomics

## Abstract

Recent studies have shown that corn-derived cationic α-D-glucan nanoparticles, known as Nano-11, significantly increase the immune response when used as a vaccine adjuvant in mice and in pigs. Furthermore, the nanoparticles can be formulated with other immunostimulators such as poly(I:C), which further enhances the immune response. The current experiments were aimed at elucidating the mechanism of action of Nano-11 alone and in combination with poly(I:C). The effect of these adjuvants on porcine monocyte-derived dendritic cells (Mo-DCs) was determined by RNA-sequencing, supplemented with flow cytometry, cytokine analysis, and Western blots. Adsorption of poly(I:C) to Nano-11 reduced its cytotoxicity for Mo-DCs. Exposure of Mo-DCs to Nano-11 and Nano-11/poly(I:C) induced differential expression of 979 and 2016 genes, respectively. Gene Ontology enrichment and KEGG pathway analysis revealed many changes in gene expression related to inflammation, innate immunity, immune response to infections, and metabolism. Nano-11 and Nano-11/poly(I:C) induced maturation of the Mo-DCs as indicated by increased expression of costimulatory molecules and MHC II. Increased expression of genes downstream of p38 MAPK activation revealed a role for this signaling pathway in the activation of Mo-DCs by the adjuvants. This was confirmed by Western blot and inhibition of TNF-secretion upon incubation with the p38 inhibitor SB203580. These experiments provide insights into the mechanism of action of the novel adjuvants Nano-11 and Nano-11/poly(I:C).

## Introduction

Commercial swine production provides high quality nutrition for the growing human population and is a major source of income for people world-wide. Control of infectious diseases through vaccinations is important from an economic and animal welfare perspective. In addition, pigs can be a source of infectious diseases such as influenza that infect humans. Swine influenza virus is one of the most common viral infections in swine and its bidirectional transmission between pigs and humans poses a public health risk (1). The development of effective vaccines against swine influenza is challenging because of the tremendous antigenic variation among circulating strains. Current commercial vaccines, administered *via* intramuscular injection, have limited effectiveness because they do not provide adequate cross-protection against variant strains. The ability to induce cross-protective immunity may be enhanced by intradermal or intranasal administration with appropriate vaccine adjuvants (2, 3). However, few adjuvants are suitable for these routes of vaccination.

We recently demonstrated that cationic α-D-glucan nanoparticles derived from sweet corn, termed Nano-11, are immunostimulatory and enhance the immune response when used as vaccine adjuvant (4, 5). Subsequent immunization studies in pigs showed that Nano-11 could be employed as an intranasal vaccine adjuvant in combination with the TLR3 agonist poly(I:C) which readily adsorbs to the positively charged nanoparticles (6, 7). Pigs inoculated intranasally with inactivated swine influenza virus in combination with Nano-11/poly(I:C) developed crossprotective antibodies and T cell responses. Additional studies have shown that Nano-11 can be utilized for intradermal vaccination in mice and pigs, resulting in a more robust immune response and potential dose-sparing compared with intramuscular immunization (8). The immunostimulatory effect of the nanoparticles was discovered empirically and the mechanism by which they enhance the immune response is incompletely understood. *In vitro* cell culture experiments demonstrated that Nano-11 induces activation of the NLRP3 inflammasome resulting in the release of IL-1β in a caspase-1 dependent manner (4, 8), but it is likely that other pathways are involved.

Dendritic cells (DCs) play a critical role in initiating and shaping the adaptive immune response to vaccines. Vaccine antigens delivered in nonlymphoid tissues are internalized and processed by dendritic cells. Under the influence of chemokines, dendritic cells migrate *via* afferent lymph vessels to the draining lymph node where they can activate antigen-specific T cells. T cell activation involves three signals: Signal 1 is provided by the T cell receptor-MHC/peptide interaction; signal 2 is the engagement of costimulatory molecules expressed by the DCs, including, but not limited to, CD80 and CD86, with their ligands on T cells; and signal 3 involves cytokines secreted by DCs that induce the differentiation of T cells into subsets with specific functions and cytokine profiles such as Th1, Th2, Th17, Tfh, and Treg (9, 10). The magnitude and quality of these signals are determined by the factors and signaling pathways that activate DCs. Different vaccine adjuvants elicit a variety of responses in DCs, thereby promoting differentiation of T cell subsets that drive distinct immune responses (11). The interaction between adjuvants and DCs is therefore critical to understanding the mechanisms by which adjuvants enhance the immune response.

Here, we used transcriptome profiling of porcine DCs in combination with flow cytometry and cytokine analysis to study the mechanisms by which Nano-11 and Nano-11/poly(I:C) activate these cells.

## Materials and methods

### Physical characterization of Nano-11 and poly(I:C)

Nano-11 was prepared as previously described (4). High molecular weight poly(I:C) was purchased from *In vivo*gen (San Diego, CA). Nano-11/poly(I:C) was prepared by simply mixing for one hour at room temperature. A zetasizer analyzer was used to measure the particle size, size distribution, and zeta potential of Nano-11 and Nano-11/poly(I:C) formulations (Malvern Panalytical Ltd, Malvern, UK).

### Generation of porcine DCs

Porcine monocyte-derived dendritic cells (Mo-DCs) were generated from the peripheral blood of 8-10 week old crossbred (Yorkshire x Landrace) pigs. Heparinized blood was diluted 1:1 with RPMI-1640 medium (Corning, Corning, NY), layered over Histopaque-1077, and centrifuged at 1400 rpm (400 x g) for 40 min at 25°C. Isolated mononuclear cells at a concentration of 25 x 10^6^ cells/ml were plated in 1 ml of pre-warmed complete RPMI (RPMI-1640 with 25 mM HEPES, 2 mM L-glutamine, 100 U/ml penicillin, 100 μg/ml streptomycin, 0.25 μg/ml amphotericin B, and 50 μM β-mercaptoethanol) containing 5% FBS in a 6 well plate overnight at 37°C with 5% CO2. The media was replaced with 2 ml complete RPMI supplemented with 25 ng/ml swine GM-CSF (#RP0940S; Kingfisher Biotech, Saint Paul, MN) and 10 ng/ml swine IL-4 (#RP0300S; Kingfisher Biotech). Every two days, 1 ml of old media was replaced with 1 ml fresh media. On day eight, immature porcine Mo-DCs were harvested by centrifugation at 1000 RPM for 5 min and resuspended at 1 x 10^6^ cells/ml.

### *In vitro* cytotoxicity assay

Porcine Mo-DCs at a concentration of 1 x 10^6^ cells/ml were resuspended in complete RPMI-1640 supplemented with 5% fetal bovine serum (FBS) and seeded in a 24-well plate with 1 ml of medium in each well. Nano-11 alone or combined with poly (I:C) was added to the wells at the indicated quantities, and the plates were incubated for 48 hours at 37°C with 5% CO_2_. The presence of cell lysis was determined using a lactate dehydrogenase (LDH) test kit (Thermo Fisher Scientific, Waltham, MA). The percent lysis was calculated as the difference between the LDH concentration in the adjuvant treated samples and the LDH concentration in cells treated only with medium, divided by the concentration of LDH in cells treated only with lysis buffer.

### Immunophenotyping by flow cytometry

Porcine Mo-DCs were seeded in a 96-well plate and treated alone or together with Nano-11 (200 µg/ml) and poly (I:C) (40 µg/ml) for 48 h at 37°C with 5% CO_2_ prior to analysis by flow cytometry. The cells were washed with Cell Staining Buffer (CSB; BioLegend, San Diego, CA) followed by blocking non-specific binding sites with 1% normal rabbit serum for 30 min in 4°C. Cells were labeled with biotinylated human CD152 (CTLA-4)-mouse IgG (Fc) fusion protein (#501-030; Ancell, Bayport, MN) followed by streptavidin-PE (#405203; BioLegend), and anti-SLA-DR-FITC (clone 2E9/13; Bio-Rad) for 45 min at 4°C. Flow cytometry was performed with an Attune NxT flow cytometer (Invitrogen, Carlsbad, CA). Data were analyzed with FlowJo software (Flowjo, Eugene, OR).

### Cytokine analysis

Porcine Mo-DCs were seeded in a 96-well plate and treated alone or together with Nano-11 (200 µg/mL) and poly (I:C) (40 µg/ml) at 37°C with 5% CO_2_. Supernatants were collected after 48 hours and analyzed by ELISA for IL-1β and TNF using commercial kits (Sigma-Aldrich, St. Louis, MO).

### Immunoblotting

Porcine Mo-DCs at a concentration of 1 x 10^6^ cells/ml were seeded in a 12-well plate with 1 ml of complete RPMI-1640. Cells were treated with 200 μg/ml Nano-11 and 40 μg/ml poly(I:C) separately or combined for 30 min; cells in media only or treated with LPS at 1 μg/ml served as controls. Cells were washed twice with cold PBS and subsequently treated with 100 μl of RIPA lysis and extraction buffer supplemented with Halt™ phosphatase and protease inhibitor cocktail (Thermo Fisher Scientific, Waltham, MA). The cell lysate was subjected to SDS-PAGE (NuPAGE™; Thermo Fisher Scientific), and proteins were transferred to a nitrocellulose membrane (BIO-RAD, Hercules, CA). The blots were air dried for 45 min and subsequently submerged in blocking buffer (SuperBlock™ T20; Thermo Fisher Scientific) with agitation for 1 hr at room temperature. Blots were then washed twice with TBS-Tween20, and probed with anti-MAPKAPK-2 (#12155; Cell Signaling, Danvers, MA), anti-phospho-MAPKAPK-2 (#3007; Cell Signaling) and anti-GAPDH (sc-47724; Santa Cruz Biotechnology, Dallas, TX). Blots were washed with fresh TBS-Tween20 three times for 10 min and incubated with goat anti-mouse or rabbit secondary antibody conjugated to horseradish peroxidase (Jackson ImmunoResearch, West Grove, PA). Chemiluminescent protein detection was obtained using Amersham ECL Prime reagent (Cytiva, Marlborough, MA), and the images were captured using a G:BOX Chemi-XRQ (Syngene, Frederick, MD).

### Messenger RNA extraction, gene expression, and transcriptome analysis

To determine the adjuvant-mediated effects on gene expression, RNA was isolated from porcine Mo-DCs after incubation for three hours with medium (negative control), Nano-11 or Nano-11/poly(I:C) using a RNeasy Plus Kit (Qiagen, Hilden, Germany). RNA screentape analysis (Agilent, Santa Clara, CA) was performed to evaluate the quality of the RNA. Only samples with an RNA Integrity Number (RIN) greater than 8 were processed for sequencing. The preparation of the RNA library for sequencing was performed in accordance with standard Illumina protocols and bioinformatics analysis was performed by Novogene (Beijing, China). Briefly, for the RNA sample preparations, a total of 0.8 - 1 µg of RNA was utilized as the starting input material for each sample. The NEBNext^®^Ultra™RNA Library Prep Kit for Illumina^®^ (NEB; New England BioLabs, Ipswich, MA) was used to construct the sequencing libraries, which were then assembled in accordance with the manufacturer’s instructions. Briefly, the enrichment of poly-A mRNA was accomplished by using poly-T oligo-attached magnetic beads to extract mRNA transcripts from the total RNA. The fragmentation process was carried out using divalent cations at high temperatures in NEBNEXT^®^ RNA First Strand Synthesis Reaction Buffer containing actinomycin D. Random hexamer primers and reverse transcriptase were used to synthesize the first strand of cDNA (M-MuLV; New England BioLabs). The second strand of cDNA was generated using DNA Polymerase I and Ribonuclease H (RNase H; New England BioLabs). The remaining overhangs were converted into blunt ends using the exonuclease and polymerase reactions. Adenylation of the 3’ ends of DNA fragments was followed by adaptor ligation (NEBNext; New England BioLabs). Purification of the library fragments was conducted using the AMPure XP system (Beckman Coulter, Indianapolis, IN) in order to choose cDNA segments that were preferably 150–200 bp in length. Purified cDNA was amplified by PCR using universal PCR primers, index primers, and high-fidelity DNA polymerase (Phusion; ThermoFisher). The AMPure XP system was used to purify the PCR products, and a bioanalyzer (Model 2100; Agilent) was utilized to evaluate the quality of the library. Index-coded libraries will undergo cluster generation and clonal amplification on a NovaSeq S2 flow cell to generate 125 bp/150 bp paired-end reads.

The sequence quality was evaluated using FastQC software. Low quality reads were filtered out by eliminating reads with a Qphred score of less than 20 in more than 50% of bases, reads with more than 10% unresolved bases, and reads containing adapters. The clean data utilized had about 97% of the reads with Q20 and 94% with Q30 (**Suppl Figure 1A**). Clean reads were mapped to the pig reference genome using HISAT2. The read counts assigned to each gene were counted using the featureCounts software program (12). To normalize gene expression levels for each gene, the fragments per kilobase of transcripts per million mapped reads (FPKM) were calculated.

The DESeq2 (Bioconductor) program was used to conduct the differential gene expression evaluation. DESeq2 employs the negative binomial distribution model to statistically determine differential gene expression. The obtained P-values were adjusted using the Benjamini and Hochberg method for multiple test correction in order to control the false discovery rate (FRD). The differentially expressed genes (DEGs) were identified by DESeq2 using an FDR threshold of 0.05. A Gene Ontology (GO) enrichment analysis of DEGs was carried out using the clusterProfiler R package. An adjusted P value of less than 0.05 was used to identify significantly enriched GO terms. Using the clusterProfiler R package, we identified statistically significant enrichment of DEGs in the Kyoto Encyclopedia of Genes and Genomes (KEGG) pathways.

The raw RNA sequencing data were deposited at the National Center for Biotechnology Information’s (NCBI) Gene Expression Omnibus (GEO) database, accession number GSE207745.

### Statistical analysis

The statistical significance of differences between experimental groups was determined by utilizing a one-way ANOVA test followed by Tukey’s multiple comparisons test using GraphPad Prism version 8.3.0 (GraphPad Software, San Diego, CA) for Windows. P values smaller than 0.05 were considered statistically significant.

## Results

### Adsorption of poly(I:C) to Nano-11 reduces cytotoxicity in porcine Mo-DCs

**Figure 1:**
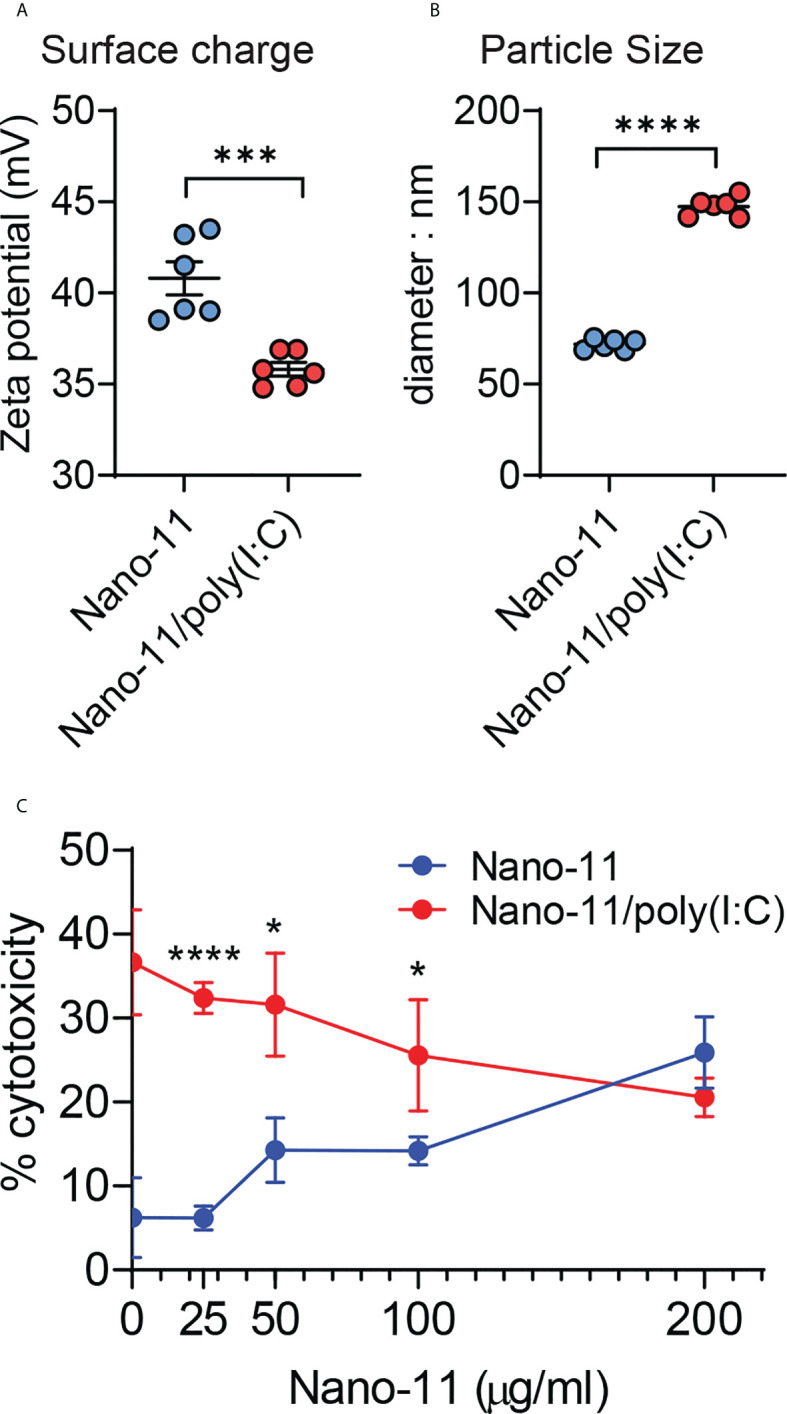
Physicochemical properties and cytotoxicity of Nano-11 and Nano-11/poly(I:C). **(A)** Zeta potential of Nano-11 and Nano-11/poly(I:C). **(B)** Particle size (diameter) of Nano-11 and Nano-11/poly(I:C). **(C)** Cytotoxicity was determined by the release of LDH from porcine Mo-DCs treated for 48 hours with Nano-11 at the indicated concentrations and combined with 40 ug/ml poly(I:C). The bars indicate the mean ± SE of three replicates. Results are representative of two independent experiments. *p < 0.05, ***p < 0.001, ****p < 0.0001.

Negatively charged poly(I:C) at 40 μg/ml adsorbs completely to 200 μg/ml cationic Nano-11 presumably *via* electrostatic interactions (6). Adsorption caused a modest decrease in the overall surface charge of the combination adjuvant (**Figure 1A**), whereas the particle size increased from 75-80 nm to 150 nm (**Figure 1B**). Poly(I:C) causes cell death *via* necroptosis in mouse bone marrow-derived DCs at 20 and 50 μg/ml (13). The viability of the porcine Mo-DCs was assessed by measuring the concentration of lactate dehydrogenase (LDH) released in the supernatant of porcine Mo-DCs incubated for 48 hours with different concentrations of Nano-11 and 40 μg/ml poly(I:C) (**Figure 1C**). Whereas poly(I:C) alone caused significant cell death with around 30-40% LDH release, this was reduced upon adsorption to Nano-11. At 200 μg/ml Nano-11 and 40 μg/ml poly(I:C), there was no difference in viability between treatment with Nano-11 alone vs Nano-11/poly(I:C).

### Nano-11 and Nano-11/poly(I:C) induce overlapping, but distinct patterns of differentially expressed genes

**Figure 2:**
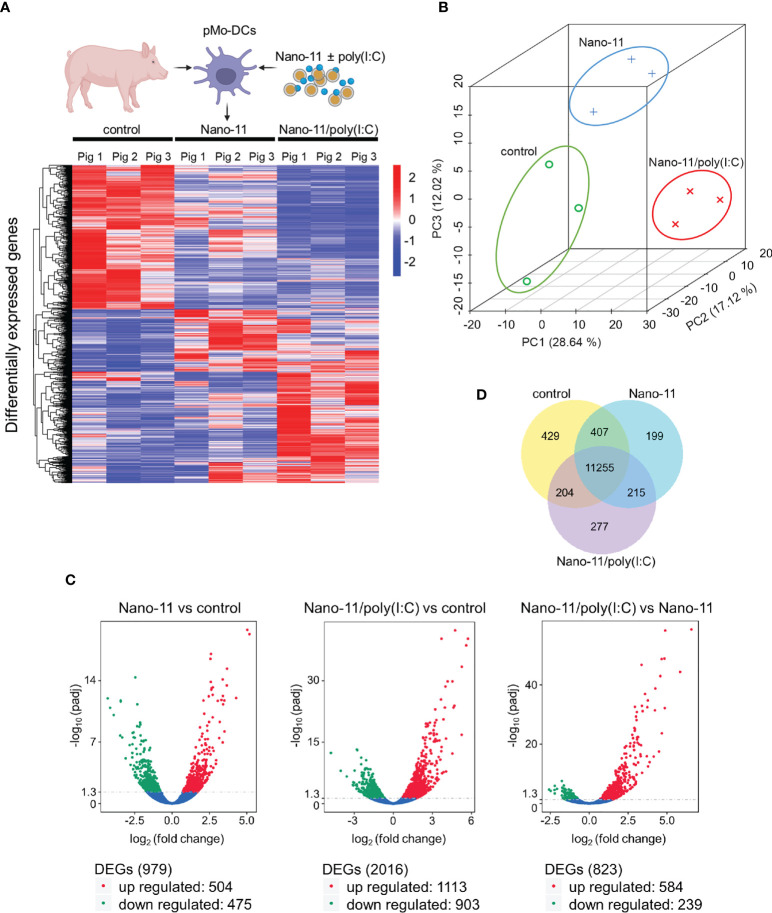
Transcriptional profiling of porcine Mo-DCs (pMo-DCs) stimulated with Nano-11 and Nano-11/poly(I:C) for 3 hours. **(A)** Hierarchical clustering heat map representing differential expression by FPKM cluster analysis. The color range indicates log10(FPKM + 1) values for high gene expression (red) levels, and genes with low expression (blue) levels. The schematic figure was created with BioRender.com **(B)** Principal component analysis (PCA) reveals clustering of DEGs from the control, Nano-11, and Nano-11/poly(I:C) groups. **(C)** Volcano plots illustrating differentially expressed genes (DEGs) after Nano-11 ± poly(I:C) stimulation. Upregulated DEGs are in red and downregulated DEGs in green. **(D)** Venn diagram depicts the number of genes that are uniquely expressed in unstimulated pMo-DCs (control), Nano-11, and Nano-11/poly(I:C) stimulated pMo-DCs, with the overlapping portions representing the number of genes that are co-expressed between the groups.

Three hours after incubation with medium only (control), Nano-11 or Nano-11/poly(I:C) mRNA was extracted from porcine Mo-DCs of three pigs. The nine libraries had an average of 67.7 million high quality reads (Supp. **Figure 1A**). Hierarchical clustering of differentially expressed genes (DEGs) using log10 (FPKM+1) revealed clusters of genes with increased and decreased levels of expression after treatment with Nano-11 alone or Nano-11/poly(I:C) compared with the control DCs (**Figure 2A**). Principal component analysis (PCA) demonstrated a clear separation of the three treatment groups based on gene expression levels (**Figure 2B**) indicating that the DCs from three different pigs produced similar gene expression patterns following treatment with Nano-11 and Nano-11/poly(I:C). We used an adjusted P-value of 0.05 to detect statistically significant differences in DEGs between the control and adjuvant treatments. This resulted in 979 DEGs for Nano-11 and 2016 DEGs for Nano-11/poly(I:C) when compared to the control (**Figure 2C**). Comparison of the two adjuvant treatments identified 823 DEGs, 239 of which were upregulated by Nano-11, and 584 by Nano-11/poly(I:C). A total of 11,255 genes were found to be expressed across all three experimental groups, while 429, 199, and 277 genes were expressed exclusively in the control, Nano-11, and Nano-11/poly(I:C) groups, respectively (**Figure 2D**).

**Figure 3:**
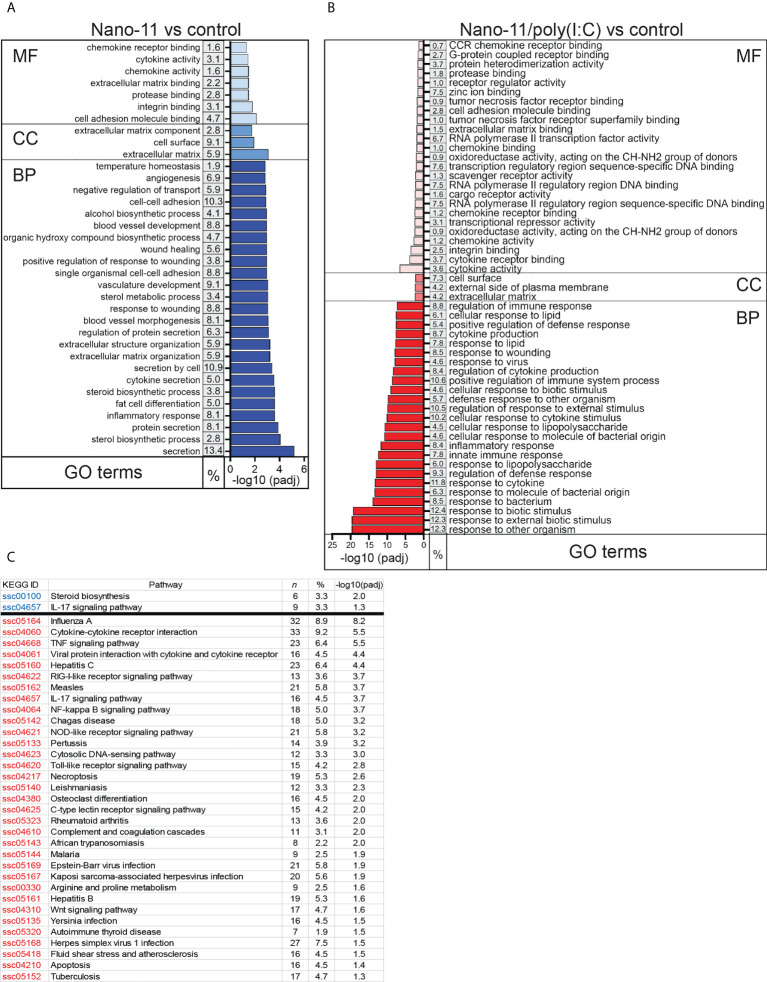
Gene ontology (GO) and KEGG pathways with the highest enrichment of differentially expressed genes (DEGs) between Nano-11 or Nano-11/poly(I:C) stimulated and untreated porcine Mo-DCs. **(A, B)** GO enrichment analysis of the DEGs following stimulation with Nano-11 **(A)** or Nano-11/poly(I:C)in comparison to untreated porcine Mo-DCs. Biological process (BP), cellular component (CC), and molecular function (MF) are GO categories. The top 25 GO terms are shown for the Nano-11 BP category and the Nano-11/poly(I:C) MF and BP categories. **(C)** KEGG pathway analysis with adjusted p-values (padj < 0.05). Significant pathways for Nano-11 are written in blue font and for Nano-11/poly(I:C) in red font. The derived P values were adjusted with the Benjamini and Hochberg method for controlling the false discovery rate by implementing the clusterProfiler R package. The gene ratio is the percentage (%) of the total number of DEGs between the GO categories or KEGG pathways and all of the DEGs listed in the GO or KEGG databases; n, the total number of DEGs that contributed to the enrichment.

Gene Ontology (GO) enrichment analysis revealed that Nano-11 significantly affected the expression of genes associated with inflammation, innate immunity, and metabolic processes (**Figure 3A**). There were seven terms associated with molecular function, three with cellular components, and 203 with biological processes. A larger number of GO terms (26 molecular function, 3 cellular components, and 440 biological processes) was identified for Nano-11/poly(I:C) including host defense responses (**Figure 3B**). Sorting of DEGs into the Kyoto Encyclopedia of Genes and Genomes (KEGG) identified only two significantly altered pathways for Nano-11, one metabolic pathway and one immune response pathway (**Figure 3C**). For Nano-11/poly(I:C), 33 KEGG pathways were identified, many of them associated with infectious diseases and immune processes. Nano-11/poly(I:C) significantly affected the expression of genes associated with the necroptosis pathway (**Figure 3**), consistent with the ability of poly(I:C) to induce this form of programmed cell death as discussed above. These results confirm the enhanced immunostimulatory activity of the combination adjuvant compared with Nano-11 alone.

### Nano-11 increases the expression of molecules associated with DC maturation

**Figure 4:**
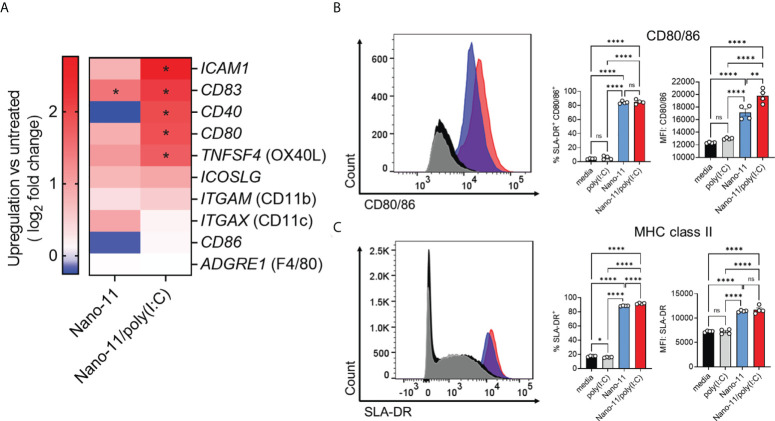
Regulation of costimulatory and MHC class II molecule expression by Nano-11 or Nano-11/poly(I:C). **(A)** Heat map comparing mRNA expression fold changes for costimulatory molecules and DC markers in porcine Mo-DCs following stimulation with Nano-11 or Nano-11/poly(I:C) compared to untreated porcine Mo-DCs. Red denotes genes with high expression levels, and blue denotes genes with low expression levels. For each experimental group the results from three different animals are shown. * indicates a significant change in gene expression at p < 0.05. **(B)** Flow cytometry analysis of porcine Mo-DC cell surface expression of costimulatory markers CD80/86 and **(C)** MHC class II (SLA-DR). The bars indicate the mean ± SE of three replicates. Results are representative of two independent experiments. *p < 0.05, **p < 0.01, ****p < 0.0001 by one-way ANOVA with Tukey multiple comparisons test. ns not statistically significant.

Incubation of Mo-DCs with Nano-11 significantly increased mRNA expression of *CD83* and caused a trend towards increased expression of *ICAM1, CD80*, *TNFSF4* (OX40L), and *ICOSLG* genes. These effects were further enhanced by Nano-11/poly(I:C) (**Figure 4A**). These molecules are associated with DC maturation and increased ability of DCs to stimulate T cells (11, 14). Nano-11 had little effect on the expression of *CD40* and *CD86*, two other genes commonly associated with DC maturation, whereas Nano-11/poly(I:C) significantly increased *CD40* gene expression. Nano-11, alone or in conjunction with poly(I:C), did not affect the expression of *ITGAM* (CD11b), or *ITGAX* (CD11c), and *ADGRE1* (F4/80).

To confirm the maturation of porcine Mo-DCs by Nano-11 and Nano-11/poly(I:C), the expression of CD80/86 and MHC II was assessed by flow cytometry. The expression of costimulatory molecules CD80/86 was determined using a CD152 (CTLA-4)–mouse IgG (Fc) fusion protein as described previously (8, 15). The expression was significantly increased upon Nano-11 treatment, and this was further enhanced by Nano-11/poly(I:C), whereas poly(I:C) alone had no effect (**Figure 4B**). The CD152 fusion protein does not distinguish between CD80 and CD86, however, the transcriptomic data suggest that Nano-11 primarily increases the expression of CD80 rather than CD86. Maturation of DCs is usually accompanied by increased expression of MHC II molecules on the cell surface (11). Indeed, incubation of Mo-DCs with Nano-11 increased the expression of MHC II molecules, while the addition of poly(I:C) had no effect (**Figure 4C**). This further confirms that Nano-11 induces DC maturation and suggests that it enhances antigen presentation by DCs.

Overall, transcriptomic analysis and expression of MHC II and CD80/CD86 indicate that Nano-11 induces maturation of porcine Mo-DCs, and this effect is amplified by the combination with poly(I:C).

### Nano-11 and Nano/poly(I:C) induce cytokine and chemokine expression in porcine Mo-DCs

**Figure 5:**
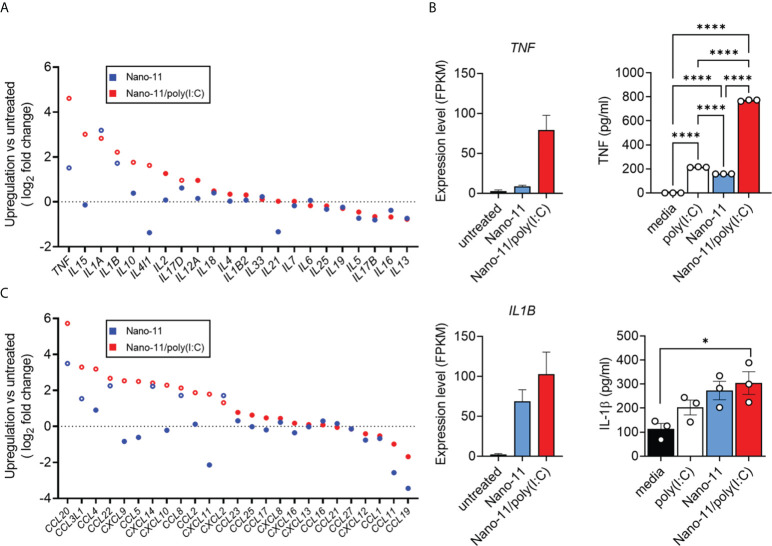
Cytokine production and expression by porcine Mo-DCs following stimulation with Nano-11 or Nano-11/poly(I:C). **(A)** Fold change of expression of cytokine-related genes. **(B)** Gene expression level of TNF and IL1B from three different animals represented by FPKM (fragments per kilobase of transcript sequence per millions base pairs sequenced). The supernatants of porcine Mo-DCs stimulated with Nano-11 ± poly(I:C) or left unstimulated as control were tested for TNF and IL-1β by ELISA. The bars indicate the mean ± SE of three replicates. Results are representative of two independent experiments. *p < 0.05, ****p < 0.0001 by one-way ANOVA with Tukey multiple comparisons test. **(C)** Fold change of expression of chemokine-related genes. Each circle represents the mean of Mo-DCs isolated from three different animals. Genes with significant mRNA upregulation (p < 0.05) are represented by open circles. The dashed line indicates no change in expression.

Nano-11 and Nano-11/poly(I:C) treatment of porcine Mo-DCs resulted in enhanced mRNA expression of pro-inflammatory cytokine genes such as *TNF, IL15, IL1A*, and *IL1B*, when compared with untreated porcine DCs (**Figure 5A**). The combination adjuvant generally induced a stronger expression of cytokine genes than Nano-11 alone. The effects on the expression of *TNF* and *IL1B* were confirmed at the protein level in the supernatants of Mo-DCs. Nano-11 and poly(I:C) each induced a modest increase of TNF, and this was markedly enhanced by the Nano-11/poly(IC) combination adjuvant (**Figure 5B**). For IL-1β, Nano-11 and the combination adjuvant induced an increase in gene expression and protein secretion without synergy between Nano-11 and poly(I:C).

Dendritic cells are potent producers of chemokines which help to orchestrate different types of immune responses (16, 17). We utilized the mRNA sequenced data to identify chemokine and chemokine receptor genes that were differently expressed in response to Nano-11 and Nano-11/poly(I:C) compared with untreated porcine Mo-DCs. The expression levels of *CCL20, CCL3L1, CCL22, CXCL14, CCL8*, and *CXCL2* mRNA were increased in porcine Mo-DCs treated with Nano-11 (**Figure 5C**). Similar to the cytokine gene expression, the expression levels were usually greater following exposure to Nano-11/poly(I:C). Furthermore, the Nano-11/poly(I:C) combination adjuvant, but not Nano-11 alone, increased upregulation of *CXCL9, CXCL10*, and *CXCL11* mRNA, in contrast to DCs treated with only Nano-11.

### Nano-11 activates the p38 MAPK signaling pathway in Mo-DCs

**Figure 6:**
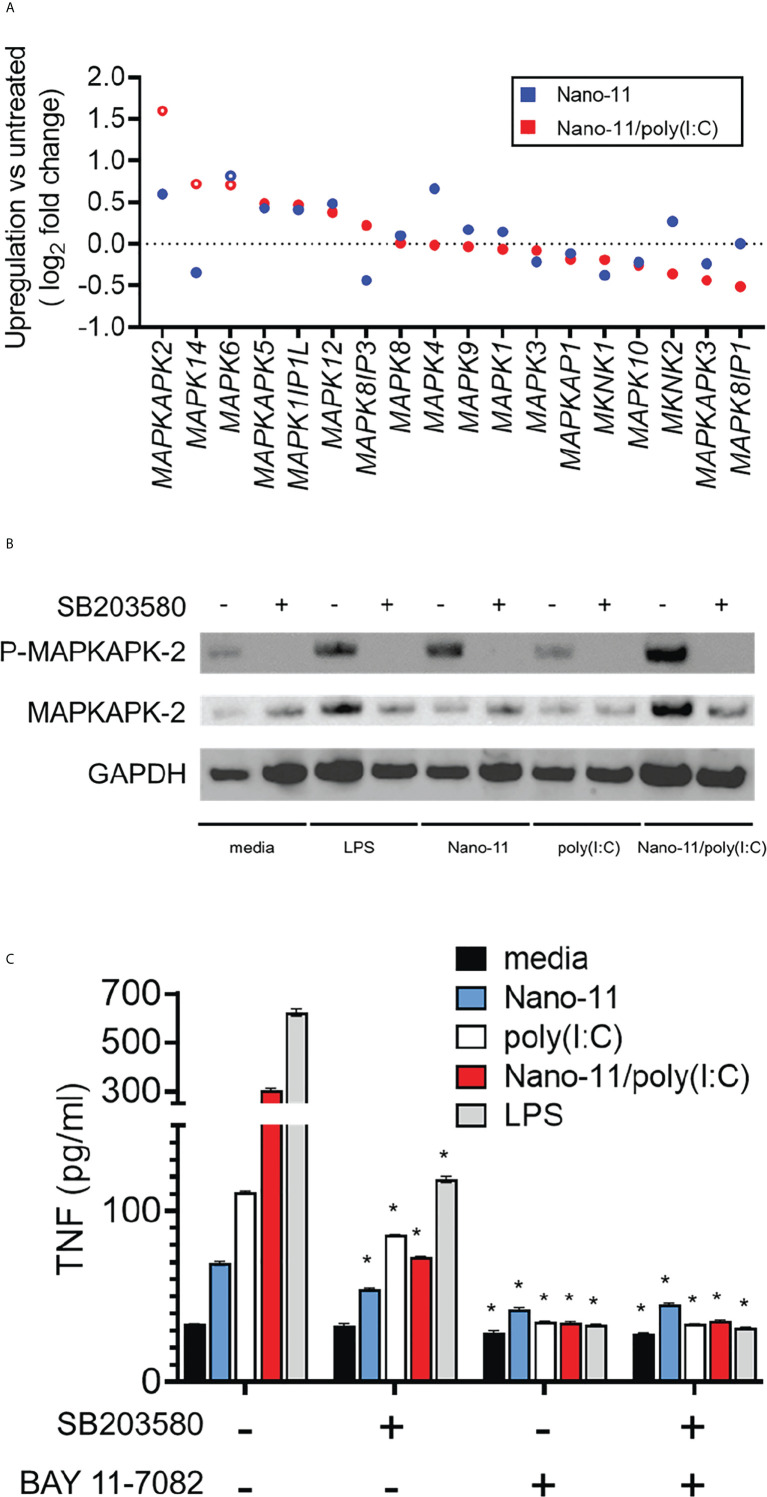
Mitogen-activated protein kinase network induced by Nano-11 ± poly(I:C). **(A)** Fold change of expression of MAP kinase family genes. No change in mRNA expression is shown by the dashed line and each dot represents the mean of three different animals. **(B)** The p38 MAPK inhibitor (SB203580) inhibits the phosphorylation of MAPKAPK2 by Nano-11 and Nano-11/poly(I:C). **(C)** Inhibition of p38 MAPK (SB203580) and NF-κB (BAY 11-7082) inhibits the secretion of TNF induced by Nano-11, poly(I:C) and Nano-11/poly(I:C). The bars indicate the mean ± SE of three replicates and statistical analysis was conducted using one-way ANOVA with Tukey test for multiple comparisons. * indicates a statistically significant effect of the inhibitor(s) on TNF secretion (p < 0.05). Results are representative of two independent experiments.

The data presented so far indicate that Nano-11 and Nano/poly(I:C) activate Mo-DCs and induce their maturation. While the receptors and signaling pathways activated by poly(I:C) are well understood (18), this is not the case for the cationic Nano-11 particles. We previously reported that Nano-11 activates the NLRP3 inflammasome, is a relatively weak activator of the NF-κB signaling pathway, and does not induce IRF signaling in human THP-1 cells (8). This suggests that other signaling pathways are involved in the activation of DCs by Nano-11. We used the RNA-sequencing data to investigate DEGs associated with mitogen-activated protein kinase (MAPK) pathways following incubation of Mo-DCs with Nano-11 or Nano-11/poly(I:C). The expression of *MAPKAPK2* was the most highly induced MAPK-related gene following treatment with the combination adjuvant and to a lesser extent with Nano-11 alone (**Figure 6A**). Nano-11 alone or with poly(I:C) also increased the expression of several other MAPK-related genes, including *MAPK4* and *MAPK14*. Here, we focused on MAPKAPK2 (MAPK-activated protein kinase 2, MK2) which is a downstream target of p38 MAPK (19, 20).

The p38 MAPK phosphorylates MK2 which is required for its activation (19). Immunoblotting on protein extracts from Mo-DCs treated with Nano-11, poly(I:C), Nano-11/poly(I:C) or LPS as a positive control showed that Nano-11 alone induced phosphorylation of MK2, whereas poly(I:C) had no effect (**Figure 6B**). Pre-treatment of the cells with the p38 inhibitor SB203580 abrogated the phosphorylation, which demonstrates that Nano-11 activates the p38 MAPK pathway. We determined the effect of the p38 inhibitor SB203580 on TNF secretion by Mo-DCs induced by Nano-11, Nano-11/poly(I:C) and LPS (**Figure 6C**). The secretion of TNF is also regulated by NF-κB, and the NF-κB inhibitor Bay 11-7082 was included to compare the relative roles of the p38 and NF-κB signaling pathways. As expected, treatment of Mo-DCs with LPS induced robust secretion of TNF, which was partially inhibited by SB203580, and completed abolished by Bay 11-7082. Nano-11 induced a modest increase of TNF, which was reduced by treatment with SB203580 and Bay 11-7082. Nano-11/poly(I:C) induced a greater level of TNF secretion which was mostly inhibited by SB203580 and completely inhibited by Bay 11-7082 (**Figure 6C**).

The involvement of p38 MAPK in the activation of porcine Mo-DCs by Nano-11 and Nano-11/poly(I:C) was further indicated by changes in the expression of genes that are dependent on p38 signaling. For example, Nano-11 increased the expression of *LIF*, *ADAMTS5* and *PTGS2* genes (encoding for leukemia inhibitory factor, a disintegrin and metalloproteinase with thrombospondin motifs 5, and cyclooxygenase-2, respectively). The expression of these genes is inhibited by treatment with the p38 inhibitor SB203580 (21–24).

## Discussion

Nano-11 enhances the immune response to vaccine antigens when administered to pigs in vaccine formulations intramuscularly, intradermally, and intranasally, however, our knowledge of the mechanisms underlying these effects is incomplete. Dendritic cells play a central role in the immunological activity of vaccine adjuvants (25). Different subtypes of DCs have been characterized in humans and in mice based on their function, phenotype, and location (26, 27). Subpopulations of DCs have also been identified and characterized in the peripheral blood and lymphoid tissues of pigs (28–30). Here, we used porcine Mo-DCs to investigate the interaction between the novel nanoparticle adjuvants Nano-11 and Nano-11/poly(I:C) with DCs through transcriptome profiling. Monocyte-derived DCs are a readily available source of DCs that can be generated from blood samples. These cells are also important in the immune response to vaccines as they infiltrate the injection site and, given the appropriate signals, transport antigens to the draining lymph nodes for presentation to T cells (31–33).

The transcriptomic data extend earlier observations indicating that Nano-11 can activate DCs directly, as indicated by the differential expression of nearly a thousand genes. Pathway analysis revealed that many of these genes are involved in innate immunity and cytokine responses as well as metabolic pathways. The combination adjuvant composed of Nano-11 with the TLR3 agonist poly(I:C) often further amplified and added to the changes in gene expression induced by Nano-11 alone which highlights the potential benefit of a combination adjuvant that activates DCs *via* multiple complementary receptors and signaling pathways.

DCs play a critical role in the initiation of immune responses by presenting antigenic peptides to naïve T cells and inducing their activation. Nano-11 induced increased expression of MHC II and several costimulatory molecules on Mo-DCs. The two best-known costimulatory molecules are CD80 and CD86. Dendritic cells constitutively express CD86 and rapidly upregulate its expression upon activation, whereas the expression of CD80 increases more slowly (34). The functions of CD80 and CD86 overlap as deletion of either molecule results in decreased Th1 and Th2 cytokine production by T cells (34). Nano-11 increased the expression of both CD80 and CD86 in human THP-1 cells and mouse dendritic cells based on phenotyping with specific antibodies (8), but increased only *CD80* gene expression in porcine DCs. Exposure of Mo-DCs to Nano-11 increased binding of the CTLA4-fusion protein, but this does not distinguish between CD80 and CD86. While the RNA data suggest that this is primarily due to increased expression of CD80, further analysis awaits the availability of specific anti-CD80 and CD86 antibodies for swine. Nano-11 increased the expression of *ICAM1*, *CD83, OX40L* and *ICOSLG* mRNA in porcine Mo-DCs. As in other species, membrane-bound CD83 plays an important role in the activation of T cells as knockdown in porcine Mo-DCs inhibits the ability of DCs to activate allogeneic T cells (35). ICAM-1 increases the strength of binding between DCs and T cells by the formation of the immunological synapse (36). OX40L is a TNF family member and ICOSLG is a member of the B7 family of costimulatory molecules. Both molecules support the induction of Th2 responses by increasing the induction and survival of Th2 cells (37, 38). The increased expression of *OX40L* and *ICOSLG* genes by Nano-11 is consistent with its ability to induce Th2-biased immune responses in mice (5).

DCs orchestrate the migration of cells critical in the immune response *via* the secretion of chemokines (16). The specific patterns of chemokine secretion induced by TLR agonists or other stimuli are reflected in functional differences (17). The expression of *CCL22* was increased upon stimulation with Nano-11 and only slightly enhanced by Nano-11/poly(I:C). The CCR4 receptor that binds CCL22 is preferentially expressed by Th2 cells and the increased expression of *CCL22* suggests that Nano-11 activates DCs to induce Th2-biased immune responses which is consistent with the roles of OX40L and ICOSL discussed above. The Nano-11/poly(I:C) combination adjuvant, but not Nano-11, induced increased expression of *CXCL9, CXCL10*, and *CXCL11* genes. These are closely related and regulated chemokines that are well known to be induced by poly(I:C) (17). The receptor for these chemokines, CXCR3, is expressed on Th1 cells, and the expression of CXCR9, -10, and -11 is a marker of Th1-inducing DCs (17). This suggests that the addition of poly(I:C) to Nano-11 will shift the immune response to a Th1-biased response. Indeed, the Nano-11/poly(I:C) combination adjuvant induced a significant increase of IgG2a antibodies in BALB/c mice compared with Nano-11 only (unpublished observations).

One of the main goals of the current study was to identify how Nano-11 alone and in combination with poly(I:C) induces DC maturation and enhances the immune response. Previous studies have shown that Nano-11 induced the secretion of IL-1β by LPS-primed dendritic cells in a caspase-1 and NLRP3-dependent manner (4, 8). Nano-11 did not activate the IRF signaling pathway and was a weak activator of the NF-κB signaling pathway based on studies with human THP-1 reporter cells (8). The ability of Nano-11 to induce expression of CD80 and several chemokine genes suggests that it can activate additional signaling pathways in DCs. Based on the differential expression of downstream genes, we identified a role for the p38 MAPK signaling pathway in the activation of Mo-DCs by Nano-11. The p38 MAPKs consist of p38α (MAPK14), p38β (MAPK11), p38γ (MAPK12) and p38δ (MAPK13) which share a high degree of homology. Of these, p38α is the most highly and widely expressed and best characterized of the p38 MAPKs (39). The p38 MAPKs are induced in response to cellular stress and inflammatory stimuli, including cytokines (39). Hence, the activation of p38 MAPK is consistent with the activation of inflammatory and metabolic processes in DCs as identified in the GO enrichment and KEGG analysis. One of the targets of activated p38 is MK2. Phosphorylation of MK2 plays a critical role in the synthesis of TNF by posttranscriptional stabilization of TNF mRNA (40). The functional relevance of p38 MAPK in the activation of Mo-DCs was demonstrated by the inhibition of TNF secretion in the presence of the p38 MAPK inhibitor SB203580. The role of p38 MAPK may differ among subpopulations of DCs as p38 activation enhanced the secretion of IL-12 in human Mo-DCs, but suppressed the secretion of IL-12 in circulating myeloid DCs (41). The authors did not examine the effect of p38 inhibition on TNF secretion by the two types of DCs.

The activation of p38 MAPK has been reported for other nanoparticles, but it does not appear to be a universal property of nanoparticles. Polystyrene and cationic lipid nanoparticles activated p38 MAPK in mouse macrophages (42, 43), whereas negatively charged silica nanoparticles inhibited p38 MAPK (44). A role for surface charge was suggested by a study in which human Mo-DCs were exposed to PLGA nanoparticles with different surface modifications (45). Only positively charged nanoparticles induced p38 MAPK activation, whereas negatively charged and neutral PLGA nanoparticles had no effect (45). These few studies suggest that one of the features of nanoparticles that drives p38 MAPK activation is a positive surface charge which is consistent with our observations with Nano-11. However, other factors such as chemical composition, size and shape, may also contribute to p38 MAPK activation.

Poly(I:C) is double-stranded RNA that can activate endosomal TLR3 and the cytoplasmic receptors retinoic acid inducible gene (RIG)-I and melanoma differentiation-associated (MDA) gene 5. The high molecular weight form, used in these experiments, preferentially activates MDA-5 over RIG-I (46). Poly(I:C) is a potent inducer of type I IFNs and it induces maturation of DCs (18, 47). However, its use as a vaccine adjuvant is limited by the potential for systemic toxicity as its receptors are broadly distributed (18). This can be avoided by adsorption or encapsulation of poly(I:C) in emulsions or particles that enhance the retention of poly(I:C) at the injection site and, preferably, target poly(I:C) to antigen-presenting cells. Previous studies have shown that Nano-11 is retained at the injection site for up to 14 days (5). Here, we show that adsorption of poly(I:C) to cationic Nano-11 enhances the activation of DCs while reducing its cytotoxicity. Therefore, the combination of Nano-11 with poly(I:C) will activate DCs *via* multiple complementary signaling pathways while reducing the potential systemic toxicity of poly(I:C).

These experiments demonstrate the power of transcriptomic analysis in elucidating mechanisms by which novel vaccine adjuvants enhance the immune response in pigs. Changes in gene expression suggest that Nano-11 induces inflammatory and metabolic changes that are associated with the activation of the p38 MAPK pathway. In addition, increased gene expression of selected costimulatory molecules and chemokines by Nano-11 are consistent with the induction of a Th2-biased response which can be shifted to a Th1 response by the addition of poly(I:C). Further studies will seek to complement these *in vitro* experiments with Mo-DCs with *in vivo* studies following vaccination with Nano-11 and Nano-11/poly(I:C) adjuvanted vaccines.

## Data availability statement

The datasets presented in this study can be found in online repositories. The names of the repository/repositories and accession number(s) can be found below: https://www.ncbi.nlm.nih.gov/, GSE207745.

## Ethics statement

The animal study was reviewed and approved by Purdue Animal Care and Use Committee.

## Author contributions

JH-F performed the experimental work and data analysis, and prepared the figures; JH-F, SX, and JT performed bioinformatic analysis; DR provided samples for analysis; HH designed and supervised the research; JH and HH wrote the manuscript. All authors contributed to the article and approved the submitted version.

## Funding

This work was supported by United States Department of Agriculture, National Institute of Food and Agriculture (USDA–NIFA) Agriculture and Food Research Initiative (AFRI) Competitive Grant 2019-67015-29814 and USDA–NIFA Hatch formula funds of project IND020164H.

## Acknowledgments

We thank Dr. Yuan Yao (Purdue University) for assistance with the preparation of Nano-11.

## Conflict of interest

The authors declare that the research was conducted in the absence of any commercial or financial relationships that could be construed as a potential conflict of interest.

## Publisher’s note

All claims expressed in this article are solely those of the authors and do not necessarily represent those of their affiliated organizations, or those of the publisher, the editors and the reviewers. Any product that may be evaluated in this article, or claim that may be made by its manufacturer, is not guaranteed or endorsed by the publisher.
