# Supplementary material for: Mechanism of activation of porcine dendritic cells by an α-D-glucan nanoparticle adjuvant and a nanoparticle/poly(I:C) combination adjuvant
Source: Front Immunol. 2022 Sep 5;13:990900. doi: 10.3389/fimmu.2022.990900 (PMC9483091; doi:10.3389/fimmu.2022.990900)
Supplement: Supplementary file 1 [file DataSheet_1.pdf]

Supplementary Information

Mechanism of activation of porcine dendritic cells by an α-D-glucan nanoparticle adjuvant and a nanoparticle/poly(I:C) combination adjuvant

Juan F. Hernandez-Franco, Shaojun Xie, Jyothi Thimmapuram, Darryl Ragland, Harm HogenEsch

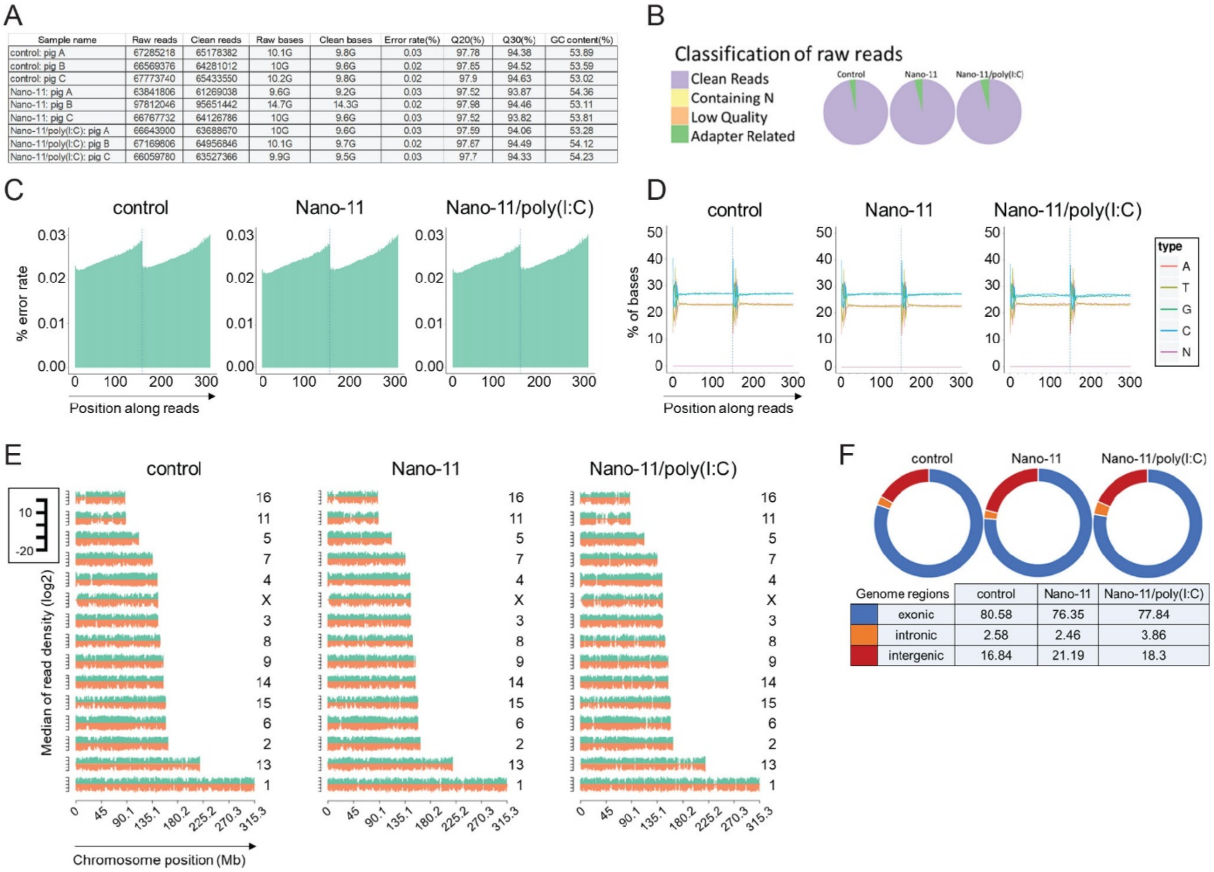

Supplementary Figure 1. RNA sequence data quality control overview. (A) Raw reads for each of the 9 samples. Clean reads represent data that has been filtered from the raw data. Raw bases were calculated by multiplying the number of raw reads by the sequence length. Clean bases is the number of clean reads multiplied by the sequence length. (B) Proportion of different categories of reads. (C) Error rate (%) of the complete sequence. Q20(%) and Q30(%) are the Phred quality scores. (D) GC content (%) is the percentage of G and C base numbers relative to the total number of bases. (E) The density distribution of reads on chromosomes. (F) The distribution of reads mapped to exonic, intronic, and intergenic regions of the reference genome.
